# Research on the Current Situation and Countermeasures of Inpatient Cost and Medical Insurance Payment Method for Rehabilitation Services in City S

**DOI:** 10.3389/fpubh.2022.880951

**Published:** 2022-06-28

**Authors:** Dongfeng Tang, Jinwei Bian, Meihui He, Ning Yang, Dan Zhang

**Affiliations:** ^1^Institute for Hospital Management, Tsinghua Shenzhen International Graduate School, Tsinghua University, Shenzhen, China; ^2^School of Economics and Management, University of Science and Technology Beijing, Beijing, China

**Keywords:** rehabilitation, bed-day payment method, hospitalization cost, rehabilitative service delivery system, three-tier rehabilitative network

## Abstract

**Objective:**

This study aimed to introduce bed-day payment for rehabilitation services in City S, China, and analyze the cost of inpatient rehabilitation services. Key issues were defined and relevant countermeasures were discussed.

**Methods:**

The data about the rehabilitation cost of 3,828 inpatient patients from June 2018 to December 2019 was used. Descriptive statistics and the Kruskal–Wallis test were employed to describe sample characteristics and clarify the comparity of cost and length of stay (LOS) across different groups. After normalizing the distribution of cost and LOS by Box–Cox transformation, multiple linear regression was used to explore the factors influencing cost and LOS by calculating the variance inflation factor (VIF) to identify multicollinearity. Finally, 20 senior and middle management personnel of the hospitals were interviewed through a semi-structured interview method to further figure out the existing problems and countermeasures.

**Results:**

(1) During 2015–2019: both discharges and the cost of rehabilitation hospitalization in City S rose rapidly. (2) The highest number of discharges were for circulatory system diseases (57.65%). Endocrine, nutritional, and metabolic diseases were noted to have the longest average length of stay (ALOS) reaching 105.8 days. The shortest ALOS was found to be 24.2 days from the diseases of the musculoskeletal system and connective tissue. Neurological, circulatory, urological, psychiatric, infectious, and parasitic diseases were observed to be generally more costly. (3) The cost of rehabilitation was determined to mainly consist of the rehabilitation fee (23.63%), comprehensive medical service fee (22.61%), and treatment fee (19.03%). (4) Type of disease, age, nature of the hospital, and grade of the hospital have significant influences both on cost and LOS (*P* < 0.05). The most critical factor affecting the cost was found to be the length of stay (standardized coefficient = 0.777). (5) The key issues of City S's rehabilitative services system were identified to be the incomplete criteria, imperfections in the payment system, and the fragmentation of services.

**Conclusions:**

Bed-day payment is the main payment method for rehabilitation services, but there is a conflict between rapidly rising costs and increasing demand for rehabilitation. The main factors affecting the cost include the length of stay, type of disease, the grade of the hospital, etc. Lack of criteria, imperfections in the payment system, and the fragmentation of services limit sustainability. The core approach is to establish a three-tier rehabilitative network and innovate the current payment system.

## Introduction

Rehabilitation allows individuals with health problems to improve their functional status and reduce disability through interventions that interact with the environment (1). Thus, improving the accessibility and affordability of rehabilitation services is essential for maintaining the population's health (2). With the accelerated aging of people and changes in the disease spectrum, the demand for rehabilitation continues to increase (3). Alarcos Cieza et al. (4) estimated that one-third of the world's population will need rehabilitation. The rapid growth in demand will inevitably create challenges for health care systems and health insurance, hence, the WHO in 2017 called to establish ten priority action areas, including the inclusion of rehabilitation in the universal health coverage, the establishment of a comprehensive rehabilitation services model, and the expansion of financing (5). Developed countries are at the forefront of the world's response to this issue (6–8). In 2002, the United States introduced a prospective payment system taking various factors, including patient diagnosis, functional status, age, and co-morbidities into consideration, to limit the cost increase and improve the quality of rehabilitation (9). Thus, a new payment system named FRGs based on the patient's functional status was developed (10). Another prospective payment system developed by GMS is Resource Utilization Groups (RUGs). Patients with similar resource utilization characteristics are divided into a group, and the case portfolio index or payment weight is calculated for each group. The facilities get payment based on their resource utilization (11). To constrain costs more effectively, Patient-Driven Payment Model (PDPM) was developed further. Patients are divided into groups according to clinical characteristics, such as main disease type and complications, and disability and dementia situations (12, 13). Australia and the UK have also developed different case groups and strategies for rehabilitation to better promote rehabilitation development (14).

In the dual context of China's comprehensive promotion of the construction of a healthy China and the implementation of a strategy to actively cope with an aging population (15), rehabilitation services are also receiving increased attention from the Chinese policymakers. The National Health Commission of the People's Republic of China pointed out the need to improve the rehabilitation services delivery system and enhance the capacity of rehabilitation services in its “Opinions on Accelerating the Development of Rehabilitation” (16). The Chinese medical insurance is a typical public contract model, so the reform in payment methods from the administration will have great leverage (17). Governments from central to local levels are exploring reasonable payment systems for rehabilitation services to achieve the dual effect of improving quality and efficiency (18–20). Currently, pay for service (PFS) is the main payment option for rehabilitation services in most provinces in China, which easily results in waste of resources and accelerating increase in costs (21). In 2017, the General Office of the central government suggested that bed-day payment could be applied to those rehabilitation services with a long length of stay and relatively stable cost (22). Zhejiang province began to set specific groups in the DRGs system for long-term rehabilitation hospitalization in 2019, taking average length of stay, average daily cost, and quality into account (23).

City S lies to the southeast coast of China, with a population of about 17.56 million in 2020. It is a relatively developed city, hospitals beds per 1,000 persons were 3.58 and physicians per 1,000 persons was 2.43. Now, it is in a dilemma where the medical resources especially quality resources are insufficient and the unmet need of residents for medical service is growing rapidly (24). The Health Security Bureau of City S Municipality (HSBS) realized the importance of rehabilitation earlier. In order to further reduce the economic burden of patients who need long-term rehabilitation, HSBS carried out a series of reforms, which could provide valuable lessons to other places. It started to sign rehabilitation service contracts with general or rehabilitative hospitals to better meet residents' rehabilitation needs in 2009. As of December 2020, HSBS has contracts with 13 hospitals: three of them are public, and ten are private, including three tertiary hospitals, three secondary hospitals, and seven primary hospitals. Patients with the following two conditions are eligible for the contract: vegetative people in a stable condition and needing long-term rehabilitative inpatient care; patients suffering from advanced tumors or cerebrovascular accident sequelae and trauma or needing hospice care (25). From 2015 to 2019, a total of 10,872 patients enjoyed this long-term rehabilitation care service.

The implementation of the program has primarily met the needs of residents for the long-term rehabilitation. Still, it is also facing the dilemma of much unmet demand from residents and excessive cost increases. Improving efficiency and quality has become a pressing problem for policymakers. This study aimed to analyze the cost of inpatient rehabilitation services in City S and discuss ways to improve the medical insurance policy and rehabilitative service delivery system.

## Materials and Methods

### Quantitative Research Methods

#### Materials

A retrospective study using data from HSBS was performed, selecting patients under the long-term rehabilitation service contracts. The information about cost, disease types (categorized by ICD 10), and length of stay (LOS) of all the discharges between 2015 and 2019 was provided by HSBS. With the help of HSBS, the data (*N* = 3,828) from June 2018 to December 2019 was extracted from the patients' electronic medical records of 13 hospitals under the contract after filtering out sensitive information (i.e., name, address). Information about sex, age, detailed cost categories, disease types (categorized by ICD 10), and other information in the medical records were included. Furthermore, the data were processed as follows: (1) exclude cases missing information about cost and length of stay; (2) exclude cases inconsistent with the actual situation, such as cases with length of stay greater than the duration from June 2018 to December 2019. Therefore, 59 cases were excluded and the final sample size was 3,769.

#### Data Analysis

The source data were entered using EXCEL 16.0 software to establish an independent database. Descriptive statistics were used to analyze the costs and sample characteristics. Due to the non-parametric distribution of data, the Kruskal–Wallis test was used to clarify the comparity of costs and length of stay in the different groups of patients; the multiple linear regression method was used to analyze the influencing factors of hospitalization cost and length of stay. Considering the hospitalization cost and length of stay were not normally distributed according to skewness and kurtosis, the Box–Cox transformation was performed. Variance inflation factor (VIF) was used to identify multicollinearity for the multivariate regression model. No VIF >10 was accepted (26). *P* < 0.05 (two-tailed) was regarded as statistically significant. All the statistical analyses were implemented using the Stata 16.0.

### Qualitative Research Methods

To know more about current situation and explore countermeasures, semi-structured interviews were conducted with the middle and senior leaders of five hospitals that had contracts with HSBS. Two of the hospitals interviewed were tertiary hospitals, one was secondary, and two were primary. Besides, three of them were public and two were private. The interviewees included the president, vice president, director of the medical insurance department, and director of the rehabilitation department, for a total of 20 people. A separate interview with each person was conducted face to face and the total time was 720 min. The main contents were about the development status of the rehabilitation business of the hospitals, opinions on the current payment method, and the problems and countermeasures of the rehabilitation system in City S. The whole interviews were recorded, and after the data were compiled and analyzed, key issues were defined.

## Results

### Sample Characteristics

Among all the 3,769 patients, 66.92% (*N* = 2,419) were male and 39.98% (*N* = 1,507) were 40–60 years old. The circulatory system diseases accounted for the largest proportion of 64.02% (*N* = 2,413). In total, 80.84% (*N* = 3,047) of patients were from private hospitals, 65.11% (*N* = 2,454) were from primary hospitals, followed by secondary hospitals with 25.76% (*N* = 971), and finally tertiary hospitals with 9.13% (*N* = 344). The cost and length of stay were found to be significantly different (*P* < 0.05) across groups of age, type of disease, nature of the hospital, and grade of the hospital (see Table 1).

**Table 1:** Sample characteristics.

	***N*** **(%)**	**Average cost**		***P*** **-value**	**Average length of stay**		***P*** **-value**
		**Mean**	**SD**		**Mean**	**SD**	
Sex				0.828			0.659
Male	2,419 (66.92%)	7,955.78	106.12		65	0.81	
Female	1,196 (33.08%)	7,993.53	154.26		65	1.12	
Age				*P* < 0.001			*P* < 0.001
0–20	105 (2.79%)	4,542.39	273.23		41	2.53	
20–40	615 (16.32%)	6,319.15	200.14		53	1.48	
40–60	1,507 (39.98%)	7,800.70	129.69		64	1.02	
60–80	1,072 (28.44%)	8,808.24	166.93		69	1.19	
>80	470 (12.47%)	8,756.73	240.79		72	1.81	
Type of disease				*P* < 0.001			*P* < 0.001
Tumors	169 (4.48%)	5,083.97	350.08		38	2.64	
Mental and behavioral disorders	25 (0.66%)	7,982.80	1,321.22		70	11.72	
Neurological diseases	328 (8.70%)	8,637.93	408.84		70	2.75	
Circulatory system diseases	2,413 (64.02%)	8,446.89	96.58		68	0.73	
Diseases of the skeletal muscle system and connective tissue	245 (6.50%)	2,648.85	96.38		21	0.86	
Injury, poisoning, and certain other results of exogenous effects	147 (3.90%)	8,491.56	342.95		77	2.86	
Factors affecting health status and exposure to health services	194 (5.15%)	6,582.26	323.08		54	2.33	
Other diseases	248 (6.58%)	8,985.65	392.60		75	2.99	
Nature of the hospital				*P* < 0.001			*P* < 0.001
Public	722 (19.16%)	5,796.73	180.93		53	1.70	
Private	3,047 (80.84%)	8,366.18	93.97		67	0.67	
Grade of the hospital				*P* < 0.001			*P* < 0.001
Primary	2,454 (65.11%)	7,373.24	97.69		64	0.77	
Secondary	344 (9.13%)	2,787.64	113.29		22	0.91	
Tertiary	971 (25.76%)	10,941.40	163.69		79	1.17	

### Cost

#### Cost From 2015 to 2019

The bed-day payment method was adopted under the rehabilitation service contract. In 2015, all facilities shared the same payment rate and were paid by 80$ per bed-day. In 2016, the payment rate began to take medical service price level into consideration. In City S, the price of inpatient medical services was divided into 3 levels: level 1, which was the standard price and applied to tertiary hospitals; level 2, 95% of level 1 and applied to secondary hospitals; level 3, 90% of level 1 and applied to primary hospitals. Therefore in 2016, the payment rate was divided into 3 classes: 105.6, 112, and 116.8 ($/bed-day). Tertiary hospitals enjoyed a higher rate than secondary or primary hospitals for the same service. The rate was adjusted dynamically and the formula was “payment rate = average inpatient cost of each bed-day in recent 3 years^*^ (1 + basic growth rate)^*^ (1 + inpatient price growth rate)^*^ (1 + price level rate)”. In 2019, the payment rates reached 130.4$ for tertiary hospitals, 123.8$ for secondary hospitals, and 117.3$ for primary hospitals (27).

During 2015–2019, the total discharges in City S for inpatient rehabilitation was 10,782 and tended to increase with an average growth rate of 166% each year. During this period, the cost of inpatient rehabilitation increased year by year with an average growth rate of 79.83%, and the growth rates in 2018 and 2019 were both over 100%. In 2015, the cost for inpatient rehabilitation accounted for and 4% of the total expenditure of the city's medical insurance fund, but it became 8.69% in 2019. The average cost per capita in 2015 was only 6,248.56$, while it rose to 7,930.864$ in 2019 (see Table 2).

**Table 2:** Cost from 2015 to 2019.

	**2015**	**2016**	**2017**	**2018**	**2019**
Inpatient rehabilitation cost (billion, $)	0.03808	0.07904	0.12592	0.15936	0.36
Growth rates	–	107.56%	59.31%	26.56%	125.90%
Share of the total medical insurance fund expenditures	4.00%	6.00%	4.45%	4.03%	8.69%
Average cost per capita ($)	6,248.56	6,681.26	6,989.26	6,965.58	7,930.86

From 2015 to 2019, 57.65% of the discharges were from circulatory system diseases (including cerebral infarction, hypertension, cerebral hemorrhage, and heart failure); neurological system diseases (namely, Alzheimer, enceph alomyelitis, hemiplegia, and epilepsy) accounted for 10.42%; musculoskeletal system and connective tissue diseases (namely, arthrosis, fracture, lumbar disc herniation, and cervical spondylosis), accounting for 6.18%. Overall, neurological, circulatory, urological, psychiatric, infectious, and parasitic diseases were observed to be generally more costly than bone and joint and sports-related diseases. In 2019, certain infectious and parasitic diseases were the costliest diseases and the average cost had reached 9,253.31$. Digestive system diseases were the least costly and the average cost was 2,491.65$ (see Table 3).

**Table 3:** Average costs for different types of diseases ($).

**Type of diseases**	**Typical diseases**	**2015**	**2016**	**2017**	**2018**	**2019**
Certain infectious and parasitic diseases	Post-tuberculous encephalopathy	6,947.92	7,123.95	5,196.07	3,208.80	9,253.31
Tumors	Lung cancer, stomach cancer, liver cancer, etc.	3,627.17	2,638.17	4,024.31	4,855.02	5,620.56
Mental and behavioral disorders	cognitive impairment	7,071.46	6,880.04	7,305.12	5,215.90	9,064.15
Neurological disorders	Alzheimer, encephalomyelitis, hemiplegia, epilepsy	5,869.18	6,389.62	7,353.16	7,586.37	8,852.50
Circulatory system diseases	Cerebral infarction, hypertension, cerebral hemorrhage, heart failure	7,184.56	7,099.43	7,677.70	7,549.30	8,527.04
Respiratory diseases	Lung infection, respiratory failure	15,100.24	8,345.45	8,034.11	6,890.39	7,773.47
Digestive system diseases	Gastrointestinal bleeding, intestinal obstruction	0.00	0.00	4,188.03	2,617.77	2,491.65
Diseases of the musculoskeletal system and connective tissue	Arthrosis, fracture, lumbar disc herniation, cervical spondylosis	2,054.28	2,617.73	2,101.95	2,427.30	3,463.80
Genitourinary diseases	Urinary tract infection, chronic renal failure	0.00	0.00	0.00	4,722.78	8,025.73
Signs, symptoms, and clinical and laboratory abnormalities not attributable to other	Difficulty swallowing, loss of consciousness, speech difficulties, fatigue	0.00	0.00	0.00	3,750.78	6,413.54
Injury, poisoning and certain other consequences of external causes	Nerve injury, bone injury, sequelae, etc.	4,559.02	8,945.54	8,816.29	6,914.22	5,582.32
Factors affecting the state of health and the institutional basis with health care	Postoperative recovery period	3,983.71	5,609.91	8,561.38	6,034.52	6,244.50
Diseases of the musculoskeletal system and connective tissue	Arthrosis, fracture, lumbar disc herniation, cervical spondylosis	4,678.45	6,449.54	6,660.24	5,939.43	8,712.82
Other		5,569.87	9,613.88	7,912.82	6,464.61	452.46

#### Cost Structure

The per capita rehabilitation cost for 3,769 patients was calculated to be 7,873.96$ and it mainly consisted of rehabilitation fee (23.66%), comprehensive medical service fee (22.61%), treatment fee (19.03%), Chinese medicine fee (12.54%), medicine fee (12.42%), and diagnosis fee (5.46%). The comprehensive medical service fee mainly contained a general treatment operation fee and a general medical service fee. Non-surgical treatment cost-dominated treatment fee. Overall, the cost for inpatient rehabilitation was mainly consisted of rehabilitation fee and service fee that reflects the labor value of medical and nursing staff. While the cost of drugs, consumables, and diagnostic fee were observed to account for a smaller proportion (see Table 4).

**Table 4:** Composition of per capita rehabilitation medical costs ($).

**Item**	**Detailed item**	**Amount**	**Percentage**
			**(%)**
Comprehensive	General medical services	568.94	7.23
medical services	General treatment operations	732.32	9.30
	Nursing	469.87	5.97
	Other	8.82	0.11
	Total	1,779.96	22.61
Diagnostics	Pathological diagnosis	0.10	0.00
	Laboratory diagnosis	243.11	3.09
	Diagnostic imaging	122.72	1.56
	Clinical diagnosis	63.69	0.81
	Total	429.62	5.46
Treatment	Non-surgical treatment items	1,488.17	18.90
	Surgical treatment	10.28	0.13
	Total	1,498.45	19.03
Rehabilitation		1,862.84	23.66
Chinese medicine		987.76	12.54
Western medicine		874.77	11.11
Traditional Chinese medicine		0.00	1.31
Blood and blood products		103.48	0.16
Consumables		0.00	1.53
Other		12.68	2.59
Total		120.29	100.00

#### Influencing Factors of Cost

The multiple linear regression analysis was performed using inpatient rehabilitation cost (transformed by the Box–Cox model) as the dependent variable. Independent variables were: age, length of stay (LOS), type of disease, nature of the hospital, grade of the hospital, and the out-of-pocket ratio of the cost. The results showed that *F* = 721.491, *P* < 0.001, and adjusted *R*^2^ = 0.713, indicating that the regression equation held and fit well. The highest VIF was 5.710 and the model waived the risk of multicollinearity (VIF > 10). LOS, tertiary hospital (compared to primary hospital), circulatory system diseases (compared to tumors), and private hospital (compared to public hospital) were all found to be positively associated with inpatient rehabilitation cost. And the out-of-pocket ratio had a negative effect on inpatient rehabilitation cost. Furthermore, the standardized coefficient of LOS is 0.777, which was the biggest one, and the Pearson's correlation between LOS and inpatient rehabilitation cost was as high as 0.836, indicating LOS was the most influencing factor (see Table 5).

**Table 5:** Multiple linear regression analysis of costs for inpatient rehabilitation.

**Variables**	**Unstandardized coefficients**		**Standardized coefficients B**	***t***	**Significance**	***B*** **(95.0%CI)**		**VIF**
	**Unstandardized coefficients B**	**SD**				**Lower limits**	**Upper limits**	
**Constant**	103.714	3.690		28.107	*P* < 0.001	−8,818.071	−1,037.243	
**Age**	0.077	0.031	0.023	2.492	0.013	47.681	112.856	1.185
**LOS**	1.280	0.015	0.777	82.657	*P* < 0.001	626.061	658.970	1.282
**Out-of-pocket ratio of costs**	−39.290	6.373	−0.052	−6.165	*P* < 0.001	16,917.466	26,522.962	1.040
Type of diseases (**ref: tumors)**								
Mental and behavioral disorders	−9.236	7.171	−0.012	−1.288	0.198	−11,966.512	3,293.346	1.174
Neurological diseases	2.098	3.318	0.009	0.632	0.527	−2,770.556	4,281.780	3.032
Circulatory system diseases	9.970	2.674	0.074	3.728	*P* < 0.001	−2,394.836	3,317.513	5.710
Diseases of the skeletal muscle system and connective tissue	−2.668	4.591	−0.010	−0.581	0.561	−5,598.449	4,157.329	4.440
Injury, poisoning, and certain other results of exogenous effects	0.494	3.839	0.001	0.129	0.898	−8,498.907	−321.162	1.915
Factors affecting health status and exposure to health services	4.798	3.498	0.016	1.372	0.170	−4,702.563	2,742.521	2.070
Other diseases	7.382	3.382	0.028	2.183	0.029	405.959	7,553.403	2.436
Nature of the hospital (ref: public hospital)								
Private school	9.633	1.936	0.059	4.976	*P* < 0.001	2,040.646	6,164.528	2.012
Grade of the hospital (ref: primary school)								
Secondary school	5.417	3.784	0.024	1.432	0.152	−727.382	7,322.108	4.116
Tertiary hospital	24.435	1.338	0.165	18.264	*P* < 0.001	10,445.272	13,445.799	1.186

### Length of Stay

#### Length of Stay of Different Types of Diseases From 2015 to 2019

The length of stay varied in different types of diseases. Endocrine, nutritional, and metabolic diseases were found to have the longest average length of stay, reaching 105.8 days. Genitourinary diseases reached 72.8 days and the LOS of neurological disorders were 71.9 days, which were relatively longer than other types of diseases. The shortest average length of stay was found to be 24.2 days from the diseases of the musculoskeletal system and connective tissue (see Table 6).

**Table 6:** Different diseases' length of stay.

**Type**	**Typical diseases**	**Average length of stay (ALOS)**
Certain infectious and parasitic diseases	Post-tuberculous encephalopathy	61.9
Tumors	Lung cancer, stomach cancer, liver cancer, etc.	41.3
Endocrine, nutritional, and metabolic diseases	Diabetes	105.8
Mental and behavioral disorders	Cognitive impairment	68.3
Neurological disorders	Alzheimer, encephalomyelitis, hemiplegia, epilepsy	71.9
Circulatory system diseases	Cerebral infarction, hypertension, cerebral hemorrhage, heart failure	68.5
Respiratory diseases	Lung infection, respiratory failure	57.2
Digestive system diseases	Gastrointestinal bleeding, intestinal obstruction	28.3
Diseases of the musculoskeletal system and connective tissue	Arthrosis, fracture, lumbar disc herniation, cervical spondylosis	24.2
Genitourinary diseases	Urinary tract infection, chronic renal failure	72.8
Congenital malformations, deformities, and chromosomal abnormalities	Cerebral hypoplasia	52.6
Signs, symptoms, clinical and laboratory abnormalities not attributable to other	Difficulty swallowing, loss of consciousness, speech difficulties, fatigue	68.4
Injury, poisoning, and certain other consequences of external causes	Nerve injury, bone injury, sequelae, etc.	62.1
Factors affecting the state of health and the institutional basis with health care	Postoperative recovery Recovery period	69.8
Other		40.8

#### Influencing Factors of Length of Stay

Length of stay (transformed by the Cox–Box model) was set as the dependent variable in the multiple regression model. Independent variables were age, out-of-pocket ratio, type of disease, nature of the hospital, and grade of the hospital. The results showed higher age, smaller out-of-pocket ratio, public, and tertiary hospital resulted bigger length of stay. Different types of diseases were all found to be associated with LOS. But, what could not be ignored was that the adjusted *R*^2^ was only 0.252, suggesting other critical factors omitted. The highest VIF was 5.595, and there was no risk of multicollinearity (see Table 7).

**Table 7:** Multiple linear regression analysis of length of stay.

**Variables**	**Unstandardized coefficients**		**Standardized coefficients B**	***t***	**Significance**	***B*** **(95.0%CI)**		**VIF**
	**Unstandardized coefficients *B***	**SD**				**Lower limits**	**Upper limits**	
**Constant**	9.970	0.503		19.826	*P* < 0.001	37.740	53.043	
**Age**	0.030	0.004	0.108	7.103	*P* < 0.001	0.176	0.306	1.166
**Out-of-pocket ratio of costs**	−4.533	0.883	−0.074	−5.135	*P* < 0.001	−39.377	−12.287	1.037
Type of diseases(ref: tumors)								
Mental and behavioral disorders	3.827	0.993	0.059	3.852	*P* < 0.001	22.505	55.997	1.170
Neurological diseases	5.407	0.454	0.288	11.920	*P* < 0.001	29.371	43.195	2.943
Circulatory system diseases	4.240	0.367	0.385	11.541	*P* < 0.001	19.246	30.358	5.595
Diseases of the skeletal muscle system and connective tissue	4.035	0.634	0.188	6.361	*P* < 0.001	15.037	35.118	4.401
Injury, poisoning, and certain other results of exogenous effects	4.923	0.529	0.180	9.305	*P* < 0.001	21.661	37.636	1.888
Factors affecting health status and exposure to health services	3.166	0.484	0.132	6.542	*P* < 0.001	10.059	24.713	2.058
Other diseases	6.038	0.461	0.283	13.103	*P* < 0.001	36.220	50.360	2.349
Nature of the hospital(ref: public hospital)								
Private school	−2.478	0.265	−0.184	−9.366	*P* < 0.001	−25.192	−17.072	1.952
Grade of the hospital(ref: primary school)								
Secondary school	−8.285	0.508	−0.451	−16.317	*P* < 0.001	−67.829	−51.321	3.848
Tertiary hospital	2.408	0.182	0.199	13.223	*P* < 0.001	13.435	18.957	1.142

### Key Issues

Of all the interviewees, 75% were male, 35% were 40–45 years old, and 30% had 15–20 working years. There were separately 40% of total interviewees from primary and tertiary hospitals, and 60% were from public hospitals (see Table 8). Key issues were defined as followed.

**Table 8:** Demographic information of interviewees.

**Characteristics**	**Frequency**	**Percent(%)**
Gender		
Female	5	25
Male	15	75
Age		
35–40	5	25
40–45	7	35
45–50	6	30
50–55	2	10
Position rank	5	25
President		
Vice president	5	25
Director of Medical Insurance Section	5	25
Director of Rehabilitation	5	25
Working years	3	15
5–10		
10–15	5	25
15–20	6	30
20–25	1	5
25–30	4	20
30 above	1	5
Hospital grade	8	40
Tertiary		
Secondary	4	20
Primary	8	40
Hospital nature	12	60
Public		
Private	8	40

Rehabilitation-related criteria were identified to be incomplete, including patient admission and discharge criteria and grading and classification criteria, which was one of the reasons for the rapid increase in cost. Currently, the admission criteria applicable to the contract were vague, without clear regulations on the type and severity of the disease. It resulted in a mixed composition of patients. A significant number of long-term care and hospice patients, less costly but having a longer stay, were also included. But in the context of bed-day payment, the insurance needed to pay the same amount according to the length of stay, which was much more than the real cost. On the other hand, some patients had long been occupying beds due to the lack of clear discharge criteria, resulting in not only a tight bed capacity but high cost. Grading and classification criteria were needed to strengthen the management of patients and provide a reference for payment. But there was a lack of unified criteria, which brought a great barrier to the refinement of payment and quality improvement.

Single bed-day payment was insufficient to reflect the real resource usage and guarantee equity. Patients with different types of diseases or with the same disease but different degrees of severity were applicable to the same payment rate. For example, patients in critical rehabilitation usually required some surgical treatment and relied on medical instruments so that the cost of whom was much more than other kinds of patients. But the long-term care patients were much less costly, which was the main source of hospitals' profits. Therefore, hospitals might face pressure of cost control or room for profit. The equity of payment would be weakened or there might be a risk of patient selection or malpractice that reduced the quality of care. Besides, considering City S is building a separate long-term care insurance, the long-term care patients will be excluded from the rehabilitation service contract. Therefore, hospitals would face greater challenges as the average cost increase greatly.

Rehabilitation service was fragmented and failed to meet the requirements of integrated care. Most rehabilitation facilities interviewed did not have referral agreements with other hospitals, which meant that patients could not get continued care. The linkage between acute care and rehabilitation was not smooth, resulting in some patients missing the best period of rehabilitation. Besides, some rehabilitation patients still had the need for acute care. But the payment of the medical insurance only covered rehabilitation services in single hospitalization, which resulted in the insufficient care for the sake of controlling cost.

## Discussion

The data from City S showed that there was a rapid growth in the number of discharges and expenditures from 2015 to 2019, which reflected the strong demand for rehabilitation and suggested that the reform successfully satisfied part of the unmet need. City S encouraged patients in need of rehabilitation services to go to secondary or primary rehabilitation facilities. Thus, there were only 18.51% of patients in tertiary hospitals and the average out-of-pocket ratio was only 8.48%. This provided a good example for other cities or countries to release tight capacity of tertiary hospitals and improve the availability of rehabilitation services. The bed-day payment was also an innovative method compared with paying for service, which was widely used in China (28). It could reduce the waste of medical resources. However, the excessive increasing rate of expenditure, less specific payment system, and fragmented services delivery system cast a doubt on the sustainability. Therefore, there is still much room for improvement.

First, the length of stay should be reasonably controlled. The results showed that the length of stay was the most important factor influencing the growth of rehabilitation expenditure, which is consistent with other studies in China (29–32). To contain the LOS and increase resources' mobilization, diagnosis-related groups (DRGs)-based payments were wildly used in the US and other high-income countries. Hospitals were paid within the predefined scale according to classifications of DRG (33). A new *per diem* inclusive payment system called the DPC/PDPS (diagnosis procedure combination/*per diem* payment system) were adopted in Japan. In total, three periods were specified for each disease along with standardized *per diem* payments for each period and the payments diminish with increasing LOS (34, 35). Italy linked reimbursement to effective stay based on the time to reach peak improvement for different groups of conditions (36, 37). A weighted blended payment model was designed for rehabilitation by Australia, which applies a mixture of the episode and *per diem* rates. The whole rehabilitation was divided into four episodes: short stay (1–3 days), low outliers, inlier range (ALOS +/– 4 days), high outliers, and every episode was attached to a separate *per diem* rate according to the resources use (38). Thus, experience from the developed countries could be taken by City S that set a predefined payment rate based on diagnosis and interventions and makes the rate diminish over LOS.

The payment system needs to be innovated to drive up efficiency. Now, worldwide health systems are increasingly moving toward payment systems based on a fixed tariff structure for each episode of treatment and case-mix classification was adopted to drive up efficiency and to contain costs (39–42). For example, medicare beneficiaries are assigned to case-mix groups (CMGs) considering the diagnosis, age, level of motor, and cognitive function (43). Medicare would pay rehabilitation facilities predetermined per discharge rates based on the CMGs and market area wages (43). Therefore, bed-day payment rates could also be adjusted by diagnosis, grade of the hospital, function, and some other factors related to resources use. Hospitals have the right to negotiate with the medical insurance agency to set the final basic payment rate. Besides, more payment methods could be adopted for different kinds of patients (39). For certain diseases with relatively stable resource usage level, a fixed tariff could be used. For simple services lasting a long time, capitation payment can be used. For some complex services, pay for service is more appropriate. Medicare also made outlier payments when a rehabilitation facility's estimated total costs for a case exceeded a cost threshold. The outlier payment for a case was equal to 80% of costs above this threshold (44). Therefore, for some rehabilitation patients with high-medical demand that are extraordinarily costly compared with the other cases, a outlier payment is also needed. The over costly medical service provided during the hospitalization could be assigned to DIP (diagnosis-intervention packet) payment, which is a novel case-mix with a global budget payment system developed by China (45). Finally, a quality-based payment system for rehabilitation services should be further explored after various standards and assessment systems being improved.

Various criteria need to be developed and insurance management should be strengthened. Clear admission and discharge criteria are necessary to increase efficiency and enhance effectiveness. For example, CMS specified 13 qualifying medical conditions for inpatient rehabilitation facilities to distinguish rehabilitation services from acute care, including stroke, spinal cord injury, amputation, etc. (44). A scientific function assessment system is also needed because it is the basis of clarifying patients' needs and providing services reasonably (37). Establishing a third-party assessment committee to independently conduct a dynamic assessment, and the results could be used in the admission and discharge criteria or to evaluate hospitals' service quality. It also could be an important reference for payment. Finally, clinical paths could be introduced to efficiently manage hospitalization schedules (46), ensuring standardization of service.

To promote the integration of the rehabilitation service, a three-tier rehabilitative network should be built, including tertiary hospitals, secondary general hospitals or stand-alone rehabilitative centers, and community health facilities or primary hospitals. Although City S made some meaningful explorations about integrated care (47), the reforms were centered in acute care with less attention paid to the rehabilitation service delivery system. The UK has developed a three-tiered model of local, district, and regional services. More specifically, Level 1 services are discrete tertiary specialized rehabilitation services. Level 2 services are discrete specialist rehabilitation services. Level 3 services are local non-specialist rehabilitation services (39). In China, the tertiary hospitals could be the regional center delivering early rehabilitative services and responsible for the training of talents. Secondary hospitals or stand-alone rehabilitative centers provide post-acute rehabilitation. Primary hospitals or community health facilities are mainly for patients in stable conditions or needing long-term care and provide continuous services. Different levels of facilities should cooperate with each other and form a rehabilitative consortium. These facilities as a whole can provide whole-life services for patients under the guidance of patient-centered philosophy on the basis of two-way referral programs. What is more, now in China there are too many patients staying in tertiary or secondary hospitals and reluctant to go to primary hospitals (31), exacerbating the strain on rehabilitative resources. The policy must guide the flow of patients to primary rehabilitation facilities. Some measures could be taken, such as increasing the reimbursement rate of primary rehabilitative facilities and so on.

## Conclusion

City S has set a good example for other low- and middle-income countries to satisfy the unmet need for rehabilitation services. It also provided evidence for how the bed-day payment method worked to drive up efficiency. But there is a conflict between rapidly rising costs and increasing demand for rehabilitation. After identifying length of stay as the most important factor affecting cost, key issues were defined about current payment method and rehabilitation service delivery system. To contain cost and drive-up efficiency, the core approaches are to establish a three-tier rehabilitative network and innovate current payment system through introducing a classification system based on diagnosis and interventions, making the payment rate diminish over LOS and biding a mixed payment system.

## Limitations and Prospects

Only service providers were interviewed in our study, while other stakeholders' opinions especially the patients, did not receive enough attention. Due to the quality of data, the longitudinal changes of fees in different categories could not be analyzed and some key factors influencing LOS were omitted. In the future, we hope to comprehensively analyze the problems of the current rehabilitation system from the perspectives of management, supply and demand sides. Besides, more factors influencing cost and LOS would be explored if high-quality data are available.

## Data Availability Statement

The datasets presented in this article are not readily available because of privacy and ethical restrictions. Requests to access the datasets should be directed to zhang.dan@sz.tsinghua.edu.cn.

## Author Contributions

DT and DZ contributed to the conception of the study. DT, MH, JB, and DZ wrote the first draft of the manuscript and collected and analyzed the samples. DZ and NY consulted on data collection and analysis. DZ reviewed the manuscript and polished it. All authors contributed to the article and approved the submitted version.

## Funding

This work was supported by Youth Fund of the National Natural Science Foundation of China (Grant No. 72004112) and Shenzhen Educational Science 2020 Annual Planning Project (Grant No. ybzz20034). This work was also supported by Shenzhen key Research Base of Humanities and Social Sciences for Social Governance and Innovation Research, and People's Livelihood & Happiness Benchmarking Study (Grant No. 202003).

## Conflict of Interest

The authors declare that the research was conducted in the absence of any commercial or financial relationships that could be construed as a potential conflict of interest.

## Publisher's Note

All claims expressed in this article are solely those of the authors and do not necessarily represent those of their affiliated organizations, or those of the publisher, the editors and the reviewers. Any product that may be evaluated in this article, or claim that may be made by its manufacturer, is not guaranteed or endorsed by the publisher.
